# Supplementary figures and images for: Geniposide and Harpagoside Functionalized Cerium Oxide Nanoparticles as a Potential Neuroprotective
Source: Int J Mol Sci. 2024 Apr 11;25(8):4262. doi: 10.3390/ijms25084262 (PMC11049985; doi:10.3390/ijms25084262)

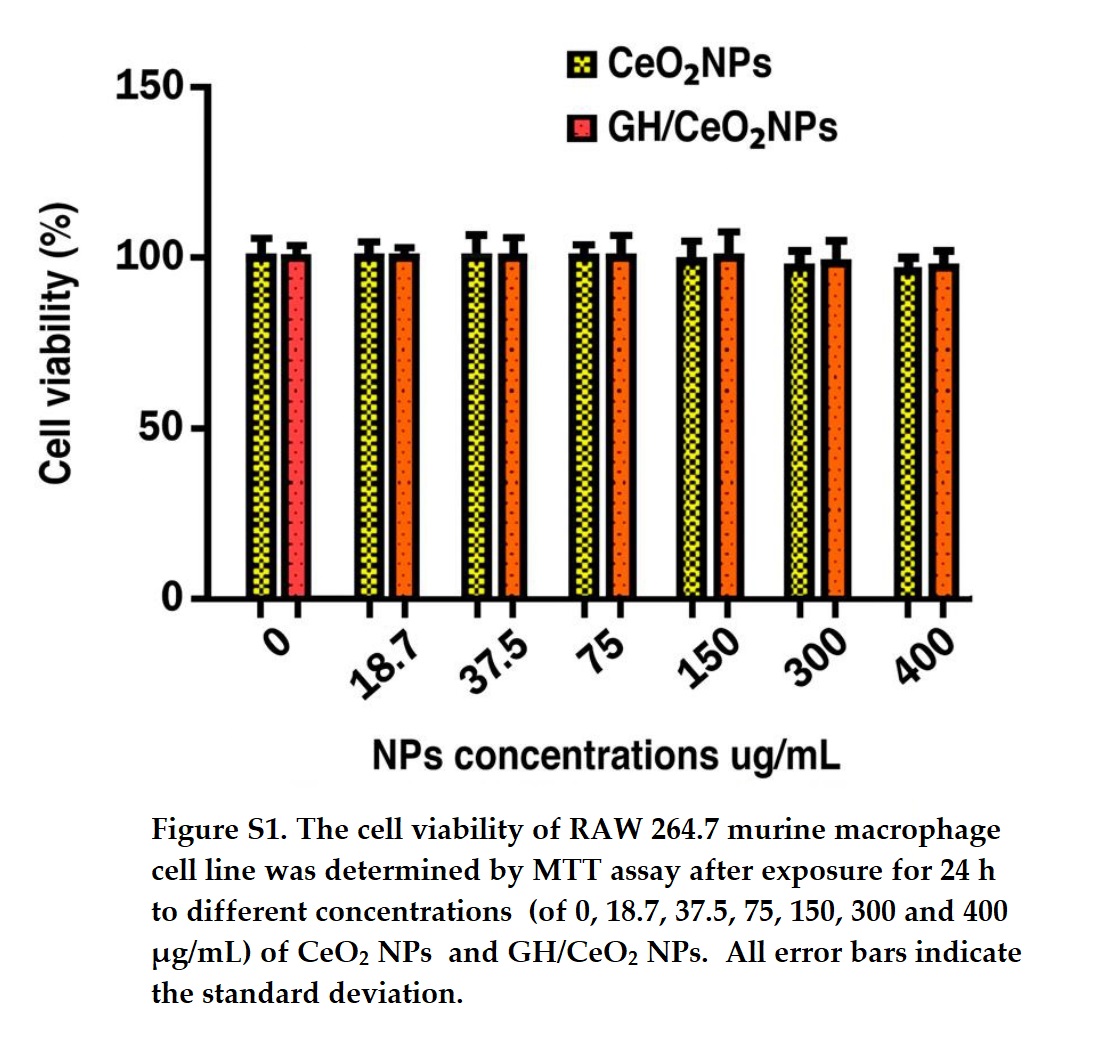

Supplement: Supplementary file 1 [file ijms-25-04262-s001.zip › ijms-2870623-supplementary.jpg]
